# Insulin-Resistance-Associated Compensatory Mechanisms of Pancreatic Beta Cells: A Current Opinion

**DOI:** 10.3389/fendo.2013.00146

**Published:** 2013-10-14

**Authors:** Tiago G. Araújo, Alexandre G. Oliveira, Mario J. A. Saad

**Affiliations:** ^1^Department of Internal Medicine, State University of Campinas, Campinas, São Paulo, Brazil

**Keywords:** HGF, betatrophin, liver, beta cells, insulin-resistance, diabetes mellitus, obesity

In obesity and in most situations of insulin-resistance, β-cells compensate for this hormonal resistance for long periods of time by an increase in secretory capacity and in β-cell mass. In animal models of insulin-resistance there is islet hyperplasia (1–3) and very recently a clear correlation between BMI and β-cells mass was shown in humans (4). The driving forces that can contribute to the increased β-cell mass in insulin-resistant states are not completely understood. It is well-established that glucose itself is able to induce β-cell hyperplasia (3, 5). However in many situations of insulin-resistance the hyperplastic response comes prior to any change in circulating glucose levels, indicating that other factors independent of glucose may contribute to the islet hyperplasia. Among circulating hormones and/or growth factors such as growth hormone (GH), insulin-like growth factor I (IGF-I), prolactin, and placental lactogen were implicated in islet hyperplasia associated with insulin-resistance. However, some data does not support the contribution of these hormones in islet hyperplasia. For example, GH and IGF-I were not altered in diet-induced obesity (DIO) mice and most insulin-resistant animal models investigated were male, making prolactin and the placental lactogen improbable candidates. In the past 4 years a novel pathway involving a neural relay and two hormones – betatrophin and hepatocyte growth factor (HGF) – were implicated as an inter-organ communication system associated to the compensatory response of β cells in face of insulin-resistance (6–8). In this commentary we will focus on evidence showing the role of this novel pathway in islet hypertrophy associated with obesity and insulin-resistance.

In a recent publication by Yi and collaborators (8), the authors identified betatrophin, a novel hormone that increases in insulin-resistant states and controls pancreatic β cell proliferation. This hormone was recognized through the infusion in mice of the insulin receptor antagonist (S961) able to induce insulin-resistance, and also provoke at a dose-dependent manner a dramatic pancreatic β cell proliferation. By microarray, they identified the hormone betatrophin from the liver and adipose tissue of these animals, which showed that it is able to induce beta cell proliferation. In addition, the authors also demonstrated that the betatrophin mRNA was increased in the liver from *ob*/*ob* and *db*/*db* mice (three to four-fold), as well as during the mice pregnancy (∼20-fold). As discussed below, HGF is a growth factor that plays a key role in regulation of islet mass increases along with hyperinsulinemia in animal models of insulin-resistance, therefore could also play a role, however, this possibility was not addressed by Yi et al. (8). Additional aspects of the work deserve further clarification, although Yi et al. showed a possible cause-effect relationship between betatrophin and an increase in pancreatic β cell proliferation in their approach, some aspects were not thoroughly clarified. For example, they did not demonstrate the correlation between circulating levels of betatrophin and the increase in islet mass; most of the experiments are in an artificial model of insulin-resistance, based on the use of an insulin receptor antagonist, and not in the traditional models of obesity and or insulin-resistance, mainly based on a diet-induced obesity (9, 10); and, the increase in mRNA of betatrophin is much higher in pregnancy than in *ob*/*ob* and *db*/*db* mice, but the increase in islet mass is usually higher in these genetic models compared to pregnancy (1, 11). In addition, the regulations of complex processes that are evolutionary conserved and/or adaptive traits, such as insulin-resistance is usually involve redundant mechanisms. In this regard, an important point we would like to emphasize is that the compensatory increase in islet cell mass and hyperinsulinemia is multifactorial and involves central nervous system (CNS) (7), and at least one more growth factor besides betatrophin, this one known as HGF, as we previously demonstrated (6).

Imai and coworkers identified a neuronal relay, originating in the liver, which enhances both insulin secretion and pancreatic β-cell proliferation (7). They showed that blocking this neural relay in rodent obesity models led to an inhibition in pancreatic islet expansion during obesity development, presenting this inter-organ communication system to be physiologically involved in compensatory β-cell proliferation. This neuronal relay is connected with signaling pathways in the liver, since it is triggered by an increase in the phosphorylation of hepatic extracellular signal-regulated kinase (ERK), which is known to be activated in the liver of a murine obesity model (12). Thus, through an adenoviral gene transduction approach that promoted a liver-selective expression of a constitutively active mutant of mitogen-activated protein kinase/ERK kinase (MEK-1), they were able to induce insulin hypersecretion and β-cell proliferation. Moreover, they also demonstrated that these pancreatic effects of hepatic ERK activation were inhibited by splanchnic afferent blockade, pancreatic vagus dissection, or midbrain transection (7).

Kahn's group has consistently showed that circulating growth factors, probably produced by the liver, are also important in the connection between insulin-resistance and the increase in islet mass (1, 2). Also, our group recently published a study (6) that presented the HGF as one of the systemic liver-derived growth factors that plays a role in insulin-resistance compensatory mechanism through the liver-to-pancreas axis in the adaptive β cell growth response. The concept of the study, initially, was that since it is well-established that the HGF is a mesenchymal-derived pleiotropic cytokine that regulates cell proliferation, anti-apoptosis, motility, and morphogenesis, suggests that HGF may be a good candidate of circulating insulin-resistance-related β-cell growth factor. Moreover, HGF has at least four characteristics that suggest a pathophysiological link between insulin-resistance and islet hyperplasia/hyperinsulinemia: (1) HGF is mainly produced by the liver; (2) it is under the regulation of the ERK pathway; (3) HGF stimulates insulin secretion and increased islet mass both *in vitro* and *in vivo*; and (4) circulating levels are elevated in obesity associated-insulin-resistance. Based on this circumstantial evidence, we studied the role of HGF in insulin-resistance compensatory mechanisms. Our approach aimed to show a possible causal relationship between an increase in circulating HGF levels and compensatory islet hyperplasia/hiperinsulinemia. In this sense, we investigated the association in a dose-dependent, longitudinal approach. Our findings showed the following: (1) there is a strong and consistent correlation between HGF and the compensatory mechanism from β-cells in three animal models of insulin-resistance; (2) that HGF increases β-cell mass in a dose-dependent manner; (3) blocking HGF shuts down the compensatory mechanisms; and (4) an increase in HGF levels seems to precede the compensatory response associated with insulin-resistance, indicating that these events occur in a causal fashion. Additionally, blockages of HGF receptor (Met) worsen the already impaired insulin-induced insulin signaling in the liver of diet-induced obesity rats.

**Figure 1:**
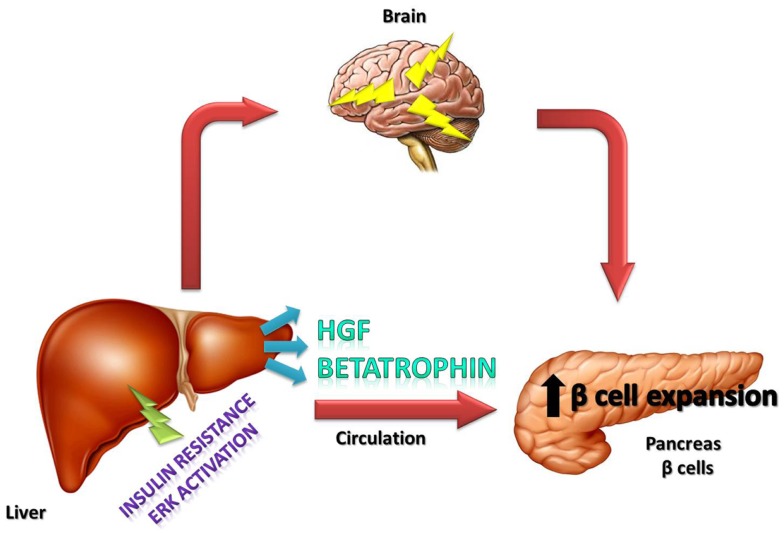
**Schematic representation of the effects of betatrophin and HGF hormones, as well as, neural circuits on islets in which these components together induce the compensatory response to insulin-resistance**.

In conclusion, it is important to emphasize that the recently described betatrophin is a hormone that has an important role in the connection between insulin-resistance and increased β-cell mass, but other growth factors such as HGF and also neural circuits certainly play an important role in this process (Figure 1). The contribution of each of these factors in different situations of insulin-resistance, such as in pregnancy, obesity, and type 2 diabetes, deserves further investigation.
